# Mitochondrial SIRT3 as a protective factor against cyclosporine A-induced nephrotoxicity

**DOI:** 10.1038/s41598-024-60453-4

**Published:** 2024-05-02

**Authors:** Ji Eun Kim, Min Jee Jo, So Yeon Bae, Shin Young Ahn, Gang Jee Ko, Young Joo Kwon

**Affiliations:** 1 Department of Internal Medicine, Korea University Guro Hospital, 148 Gurodong-ro, Guro-gu, Seoul, 08308 South Korea; 2 Department of Internal Medicine, Korea University College of Medicine, Seoul, South Korea

**Keywords:** Medical research, Pathogenesis, Nephrology

## Abstract

Sirtuin3 (SIRT3), a mitochondrial deacetylase, has been shown to be involved in various kidney diseases. In this study, we aimed to clarify the role of SIRT3 in cyclosporine-induced nephrotoxicity and the associated mitochondrial dysfunction. Madin-Darby canine kidney (MDCK) cells were transfected with Flag-tagged SIRT3 for SIRT3 overexpression or SIRT3 siRNA for the inhibition of SIRT3. Subsequently, the cells were treated with cyclosporine A (CsA) or vehicle. Wild-type and SIRT3 knockout (KO) mice were randomly assigned to receive cyclosporine A or olive oil. Furthermore, SIRT3 activator, honokiol, was treated alongside CsA to wild type mice. Our results revealed that CsA treatment inhibited mitochondrial SIRT3 expression in MDCK cells. Inhibition of SIRT3 through siRNA transfection exacerbated apoptosis, impaired the expression of the AMP-activated protein kinase-peroxisome proliferator-activated receptor gamma coactivator 1 alpha (AMPK-PGC1α) pathway, and worsened mitochondrial dysfunction induced by CsA treatment. Conversely, overexpression of SIRT3 through Flag-tagged SIRT3 transfection ameliorated apoptosis, increased the expression of mitochondrial superoxide dismutase 2, and restored the mitochondrial regulator pathway, AMPK-PGC1α. In SIRT3 KO mice, CsA treatment led to aggravated kidney dysfunction, increased kidney tubular injury, and accumulation of oxidative end products indicative of oxidative stress injury. Meanwhile, SIRT3 activation in vivo significantly mitigated these adverse effects, improving kidney function, reducing oxidative stress markers, and enhancing mitochondrial health following CsA treatment. Overall, our findings suggest that SIRT3 plays a protective role in alleviating mitochondrial dysfunction caused by CsA through the activation of the AMPK-PGC1α pathway, thereby preventing further kidney injury.

## Introduction

Calcineurin inhibitors have significantly improved graft survival in kidney transplant recipients and have played an important role in ensuring that kidney transplants are in place as an effective treatment for patients with end-stage renal failure^1,2^. Recent studies reported that majority of kidney transplant recipients maintained immunosuppressive regimen containing calcineurin inhibitors after kidney transplantation^3^. Cyclosporine A (CsA), one of the commonly used calcineurin inhibitor, has concerns about its nephrotoxicity, although its popularity for potent effect in organ transplantation as well as autoimmune diseases^4^. Chronic CsA nephrotoxicity not only deteriorates long term graft survival and patients survival, but also increases morbidity after organ transplant^4,5^. The precise mechanisms underlying CsA nephrotoxicity are not yet fully understood, but mitochondrial dysfunction-related apoptosis, induced by hemodynamic abnormalities and direct tubular injury, is considered a key contributor^6^.

Sirtuins, a family of nicotinamide adenine dinucleotide (NAD +)-dependent histone deacetylases (HDACs), play essential roles in a wide range of physiological processes, including cell survival, apoptosis, metabolism, stress responses, cancer, and aging^7–9^. Among the sirtuin family members, Sirtuin3 (SIRT3) is the most well-characterized mitochondrial deacetylase, primarily localized within the mitochondria^10^. It exerts regulatory functions by targeting and deacetylating enzymes involved in cellular metabolism, including those participating in fatty acid oxidation, the tricarboxylic acid cycle, oxidative phosphorylation, and antioxidant defense pathways^11,12^. Through its deacetylase activity, SIRT3 modulates the activity of these enzymes, promoting cellular energy homeostasis and mitochondrial function^10^. Dysregulation of SIRT3 has been implicated in various diseases, including cardiovascular diseases, neurodegenerative disorders, metabolic diseases, and cancer^11–16^.

In this study, our aim was to elucidate the mitochondrial-related mechanisms underlying CsA nephrotoxicity and evaluate the role of SIRT3 in this context. By investigating the impact of CsA on mitochondrial function and exploring the effects of SIRT3 modulation, we sought to uncover the potential of SIRT3 as a therapeutic target for mitigating CsA-induced kidney injury.

## Results

### CsA treatment inhibits mitochondrial SIRT3 expression in kidney tubular epithelial cells

**Figure 1:**
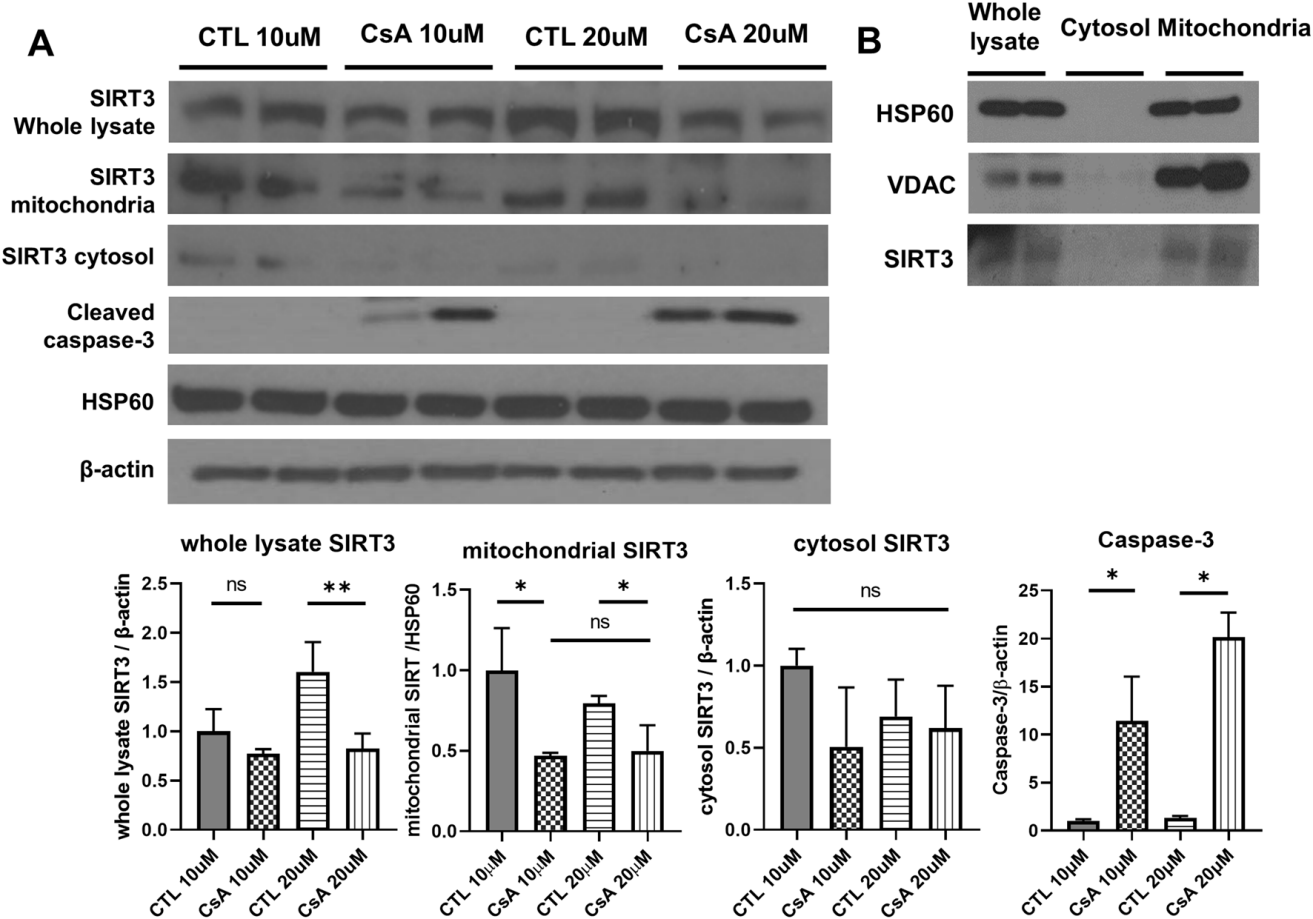
The expression of mitochondrial SIRT3 and apoptotic marker was affected by CsA administration on kidney tubular cells. (**A**) Representative blots and densitometry analyses of western blot for mitochondrial SIRT3 and cleaved caspase-3 after two different concentration of CsA injection (10 uM and 20 uM) was shown. Protein expression was normalized to HSP60 for mitochondrial SIRT3 and β-actin for whole lysate SIRT3, cytosol SIRT3 and cleaved caspase-3. (**B**) Representative blots for SIRT3, HSP60 and VDAC to examine cellular localization of SIRT3 expression. Each blot has been cropped from different gels; uncropped gels/blots are presented in Supplementary Fig. 2. Error bars represent standard deviation. Statistical significance was assessed using Kruskal–Wallis followed by Dunn’s multiple comparison test, where ns indicates not significant, and *p < 0.05 indicates statistical significance.

Cultured kidney tubular epithelial cells were treated with CsA at two different dosages to determine the dose-related effect. Compared to the control group treated with cremophor, the expression of SIRT3 in the total lysate decreased only in cells treated with 20uM CsA. However, the expression of mitochondrial SIRT3 significantly decreased in cells treated with both 10uM and 20uM CsA. CsA-treated cells, regardless of the dosage, exhibited higher expression of cleaved caspase-3, indicating increased apoptosis (Fig. 1A). In addition to assessing the dose-related effect of CsA on SIRT3 expression, we also aimed to clarify the cellular localization of SIRT3 expression by examining its presence alongside mitochondrial markers HSP60 and VDAC. Our findings confirm that SIRT3 expression is observable in the mitochondria and whole lysate, with no expression detected in the cytosol (Fig. 1B).

### Inhibition of SIRT3 aggravates CsA induced apoptosis and deteriorate the expression of peroxisome proliferator-activated receptor gamma coactivator 1 alpha (PGC1 α).

**Figure 2:**
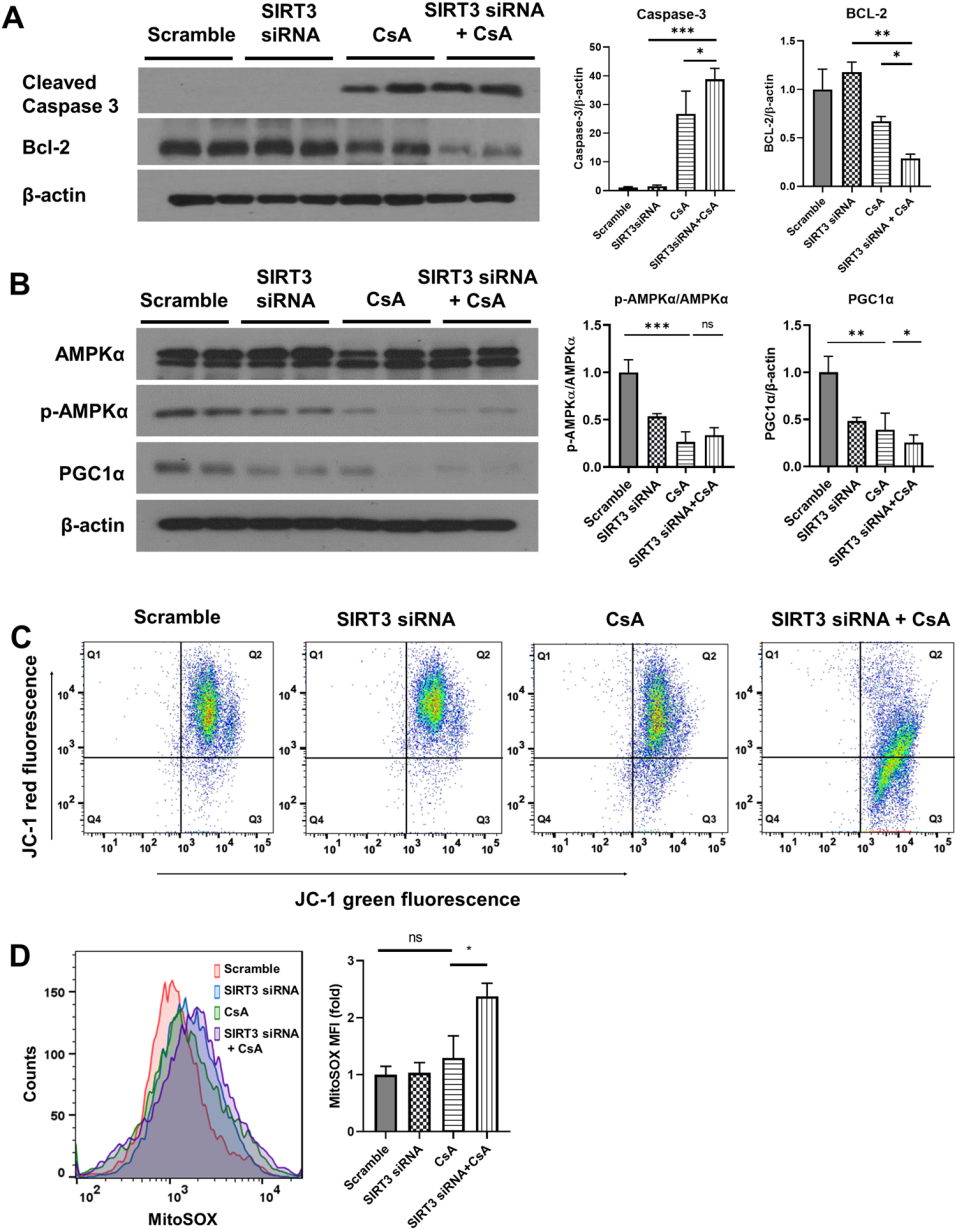
SIRT3 siRNA transfection aggravated apoptosis and mitochondrial dysfunction after CsA injury. Representative blots and densitometry of blots for apoptosis markers including caspase-3 and Bcl-2 (**A**) and mitochondrial regulatory proteins, PGC1α and p-AMPKα (**B**), were shown. Protein expressions of caspase-3, Bcl-2 and PGClα were normalized against β-actin, while p-AMPKα expression was normalized to total AMPKα. (**C**) The intensity of JC-1 fluorescence observed via flow cytometry was used as an indicator of mitochondrial membrane potential. (**D**) The mitochondrial superoxide level in cells were estimated flow cytometrically using fluorescent MitoSOX Red dye and the total superoxide levels obtained from experiment were quantitated and represented in a bar chart. Each blot has been cropped from different gels; uncropped gels/blots are presented in Supplementary Fig. 3. Error bars represent standard deviation. Statistical analysis was performed using Kruskal–Wallis followed by Dunn’s multiple comparison test. Analysis was based on three biological replicates. *ns* not significant; *p < 0.05.

The knockdown of SIRT3 using siRNA resulted in the exacerbation of CsA-induced apoptosis, as evidenced by an increase in the expression of cleaved caspase-3 and a decrease in the expression of Bcl-2 (Fig. 2A). To better understand the mechanism underlying apoptosis and injury associated with SIRT3 modulation, the changes in regulators of mitochondrial energy metabolism, namely AMP-activated protein kinase (AMPK) and peroxisome proliferator-activated receptor gamma coactivator 1 alpha (PGC1α), were investigated in CsA-induced toxicity following SIRT3 knockdown. The expression levels of phosphorylated AMPK (p-AMPK) remained unchanged with SIRT3 siRNA transfection, whereas PGC1α levels significantly decreased following CsA treatment (Fig. 2B). These findings suggest that the downregulation of SIRT3 exacerbates CsA-induced apoptosis and leads to a decrease in the expression of AMPK and PGC1α, which are important regulators of mitochondrial energy metabolism.

### Inhibition of SIRT3 exacerbates mitochondrial dysfunction induced by CsA treatment

To investigate the specific effect of SIRT3 on mitochondria in an injured state, mitochondrial function tests were conducted using two different methods. CsA treatment led to the depletion of mitochondrial membrane potential in kidney cells, and this effect further deteriorated with SIRT3 siRNA transfection (Fig. 2C). Moreover, CsA treatment increased the levels of reactive oxygen species (ROS) in the mitochondria, and the knockdown of SIRT3 exacerbated this effect (Fig. 2D). These findings underscore SIRT3's pivotal role in mitochondrial function, demonstrating its ability to alleviate kidney cellular toxicity caused by CsA.

### Overexpression of SIRT3 ameliorates CsA-induced apoptosis, increases the expression of mitochondrial superoxide dismutase 2 and restores the mitochondrial regulator pathway, AMPK-PGC1α

**Figure 3:**
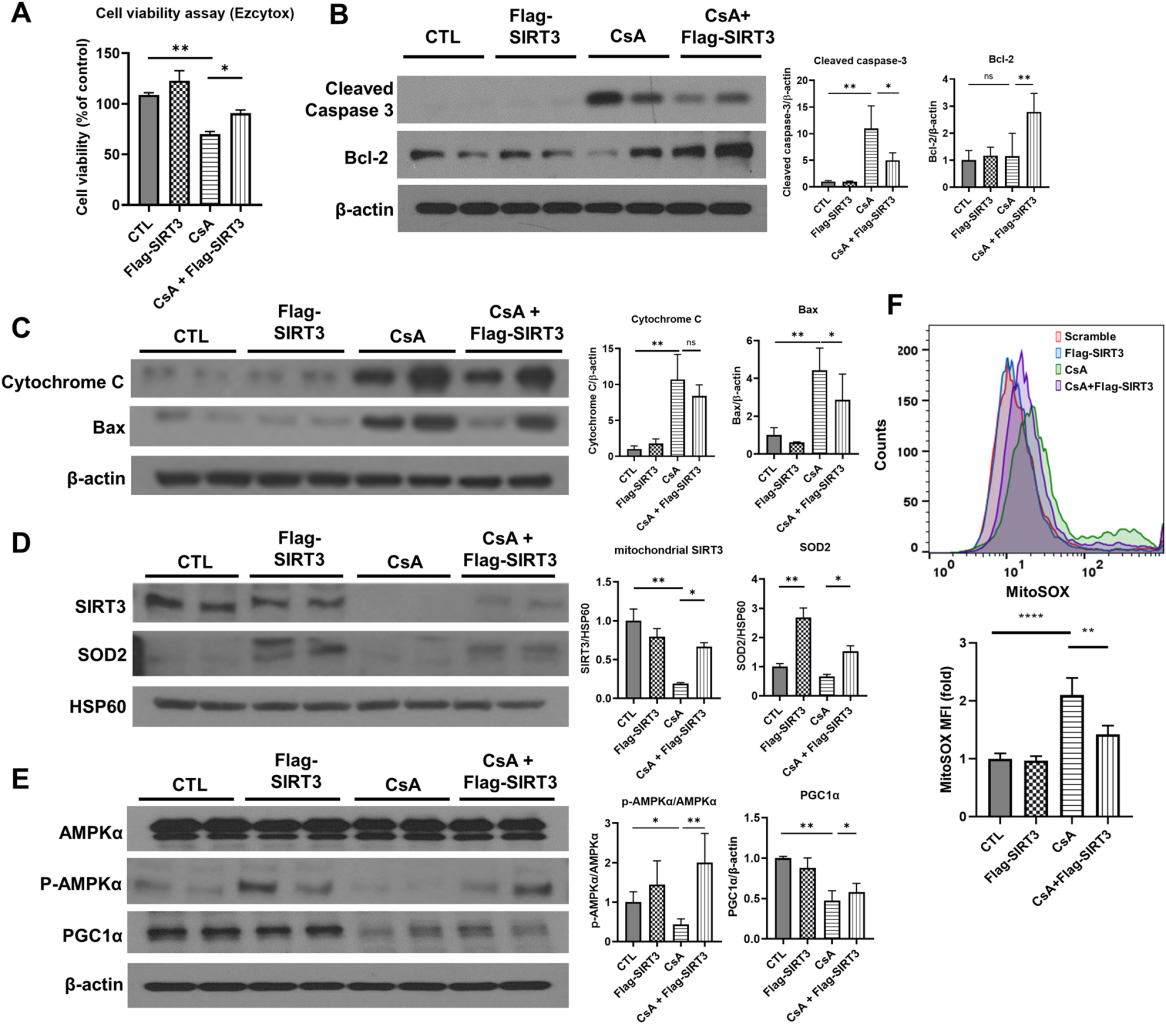
SIRT3 overexpression restored cell viability and mitochondrial SOD2 expression as well as alleviated apoptosis after CsA injury. (**A**) Cell viability was analyzed using the MTS assay. Error bars represent standard deviation. *p < 0.05, **p < 0.01. (**B**,**C**) Representative blots and densitometry of blots were shown for cleaved caspase-3, Bcl-2, cytochrome C and Bax. Protein expressions were normalized to β-actin. (**D**) Western blot analysis was performed for SIRT3 and SOD2 in mitochondrial fraction. The representative blots of independent experiments are shown on left, and the densitometry of these blots are shown on right. Protein expressions were normalized to HSP60. *p < 0.05, **p < 0.01 (**E**) A representative western blot (left) and densitometry (right) of AMPKα, p-AMPKα and PGC1α expression in whole lysate. Protein expressions of PGC1α were normalized to β-actin, and p-AMPKα to total AMPKα. (**F**) Representative histogram of MitoSOX fluorescence intensity (upper) and quantitated total superoxide levels plotted on a bar chart (lower). Each blot has been cropped from different gels; uncropped gels/blots are presented in Supplementary Figs. 4 and 5. Significance levels marked as *p < 0.05, **p < 0.01, ****p < 0.0001 based on Kruskal–Wallis followed by Dunn’s multiple comparison test, from three independent experiments.

To further validate the protective effect of SIRT3, it was investigated whether overexpression of SIRT3 could alleviate kidney toxicity induced by CsA. After SIRT3 overexpression, cell viability, which had decreased to 70.1 ± 2.5% due to CsA treatment, was significantly restored to 90.8 ± 3.0% (Fig. 3A). Western blot analysis revealed that CsA treatment increased cleaved caspase-3 levels, which SIRT3 overexpression significantly reduced. Bcl-2 levels, stable under CsA, increased with SIRT3, suggesting a enhanced anti-apoptotic effect (Fig. 3B). The expression levels of cytochrome C and Bax, also analyzed in the context of apoptosis, followed a similar pattern to cleaved caspase-3, increasing with CsA treatment and subsequently decreasing with SIRT3 overexpression (Fig. 3C). Since mitochondrial function was closely related to CsA-induced injury in kidney cells, the levels of mitochondrial Superoxide dismutase 2 (SOD2), a regulator of reactive oxygen species, were evaluated in response to SIRT3 overexpression. Following SIRT3 gene transfection, mitochondrial SOD2 levels significantly increased not only under stable conditions but also after CsA treatment (Fig. 3D). Furthermore, the regulatory proteins of mitochondrial metabolism, including PGC1α and AMPK, were reassessed, and the series of related proteins were restored by SIRT3 overexpression after CsA toxicity (Fig. 3E). We further evaluated the changes in mitochondrial ROS levels as indicated by MitoSOX fluorescence intensity. Mitochondrial ROS increased after CsA treatment but notably decreased following SIRT3 overexpression (Fig. 3F).

### Deteriorating effects of SIRT3 KO on oxidative stress and kidney injury in CsA nephrotoxicity mouse model

**Figure 4:**
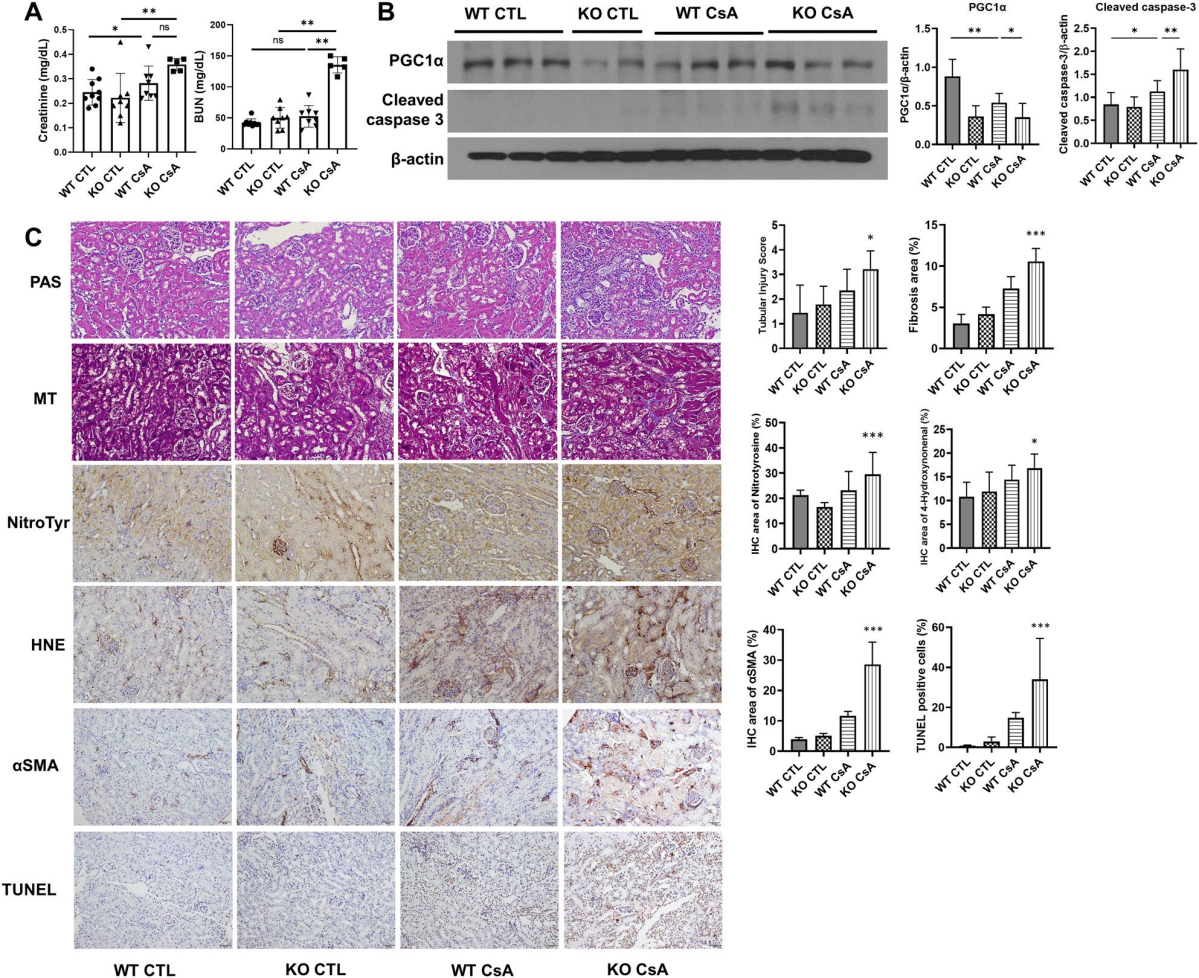
SIRT3 KO mice demonstrated exacerbated kidney dysfunction and aggravated oxidative end products compared to wild type mice in CsA nephrotoxicity. (**A**) Kidney function of mice were analyzed by serum creatinine and blood urea nitrogen levels. (**B**) Representative blots for Cleaved caspase-3 and PGC1α in kidney tissues, normalized against β-actin. Each blot has been cropped from different gels; uncropped gels/blots are presented in Supplementary Fig. 6. (**C**) Periodic acid–Schiff stain, Masson's trichrome stain, and immunohistochemical stains for nitrotyrosine, hydrononenal, αSMA and TUNEL in kidney tissues from wild type and SIRT3 KO mice after CsA injury, shown as representative images. Original magnification,  × 200. Scale bars: 100 µm. On the right side of images, bar plots for quantifying tubular injury score and the extent of staining are presented. Error bars represent standard deviation. *ns* not significant, *p < 0.05, **p < 0.01, ***p < 0.001. *WT* wild type, *KO* SIRT3 knockout, *CTL* control, *CsA* cyclosporine A.

To gain further insight into the beneficial role of SIRT3 in CsA-induced kidney cell injury through mitochondrial regulation, we evaluated changes in kidney function, histopathology, and oxidative stress marker expressions following SIRT3 expression in a mouse model of CsA nephrotoxicity. By comparing the levels of serum creatinine and blood urea nitrogen between age- and weight-matched groups of mice, CsA-treated SIRT3 knockout (KO) mice exhibited the highest levels among the groups. Specifically, serum blood urea nitrogen levels in CsA-treated SIRT3 KO mice were significantly higher compared to CsA-treated wild-type controls, while serum creatinine levels did not show a significant difference (Fig. 4A). Consistent with the in vitro analysis, kidney tissues from SIRT3 KO mice showed significantly higher expression of cleaved caspase 3 and decreased expression of PGC1α after four weeks of CsA injury compared to wild-type (WT) mice (Fig. 4B). Histological and immunohistochemical analyses were conducted to assess the impact of CsA treatment on kidney injury and oxidative stress, focusing on the exacerbating effects observed in SIRT3 knockout mice. Periodic Acid-Schiff (PAS) staining quantified tubular injury, while Masson's Trichrome (MT) staining measured fibrosis. Additionally, alpha smooth muscle actin (α-SMA), terminal deoxynucleotide transferase dUTP nick end labeling (TUNEL), 3-nitrotyrosine, and 4-hydroxynonenal stains were employed for further investigation (Fig. 4C). Compared to control mice, CsA-treated wild-type (WT) mice exhibited significantly higher tubular injury scores (0.5 ± 0.5 vs. 1.9 ± 0.7, respectively, p < 0.001), which were even more pronounced in SIRT3 knockout (KO) mice following CsA treatment (1.9 ± 0.7 in WT vs. 2.8 ± 0.7 in KO, p = 0.02). Both MT and α-SMA staining indicated a significant increase in fibrosis in SIRT3 KO kidneys post-CsA injury. The intensity and extent of staining for oxidative stress markers, 4-hydroxynonenal and 3-nitrotyrosine, were markedly elevated in the tubulointerstitial areas of SIRT3 KO kidneys, indicating severe oxidative damage. Similarly, TUNEL staining revealed a significant increase in apoptosis within the SIRT3 KO kidneys after CsA exposure, underscoring the critical protective role of SIRT3 against CsA-induced nephrotoxic effects.

### Protective effects of SIRT3 activation by honokiol in CsA-induced nephrotoxicity mouse

**Figure 5:**
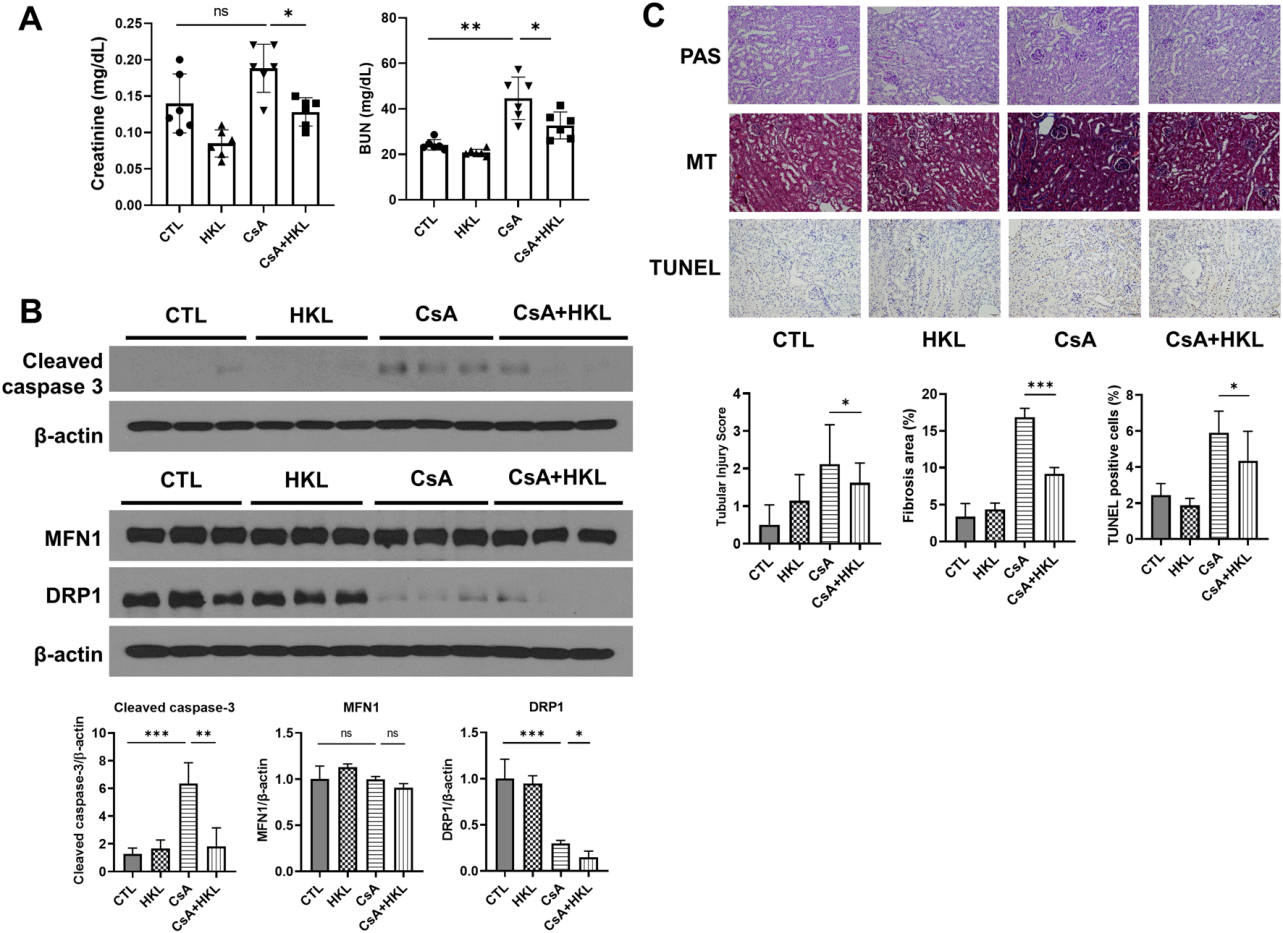
The administration of a SIRT3 activator, honokiol, alleviated CsA nephrotoxicity in mice. (**A**) Bar graphs of serum creatinine and blood urea nitrogen levels, providing a quantitative assessment of kidney function. (**B**) Representative blots for cleaved caspase-3 and genes associated with mitochondrial dynamics, MFN1 and DRP1, along with corresponding bar plots for densitometric analysis of protein expression. Protein expressions were normalized against β-actin. Each blot has been cropped from different gels; uncropped gels/blots are presented in Supplementary Fig. 7. (**C**) Representative images of Periodic Acid-Schiff, Masson's trichrome, and TUNEL staining of kidney tissues, with subsequent bar plots quantifying the stained areas. Original magnification, × 200. Scale bars: 100 µm. Each blot has been cropped; full-length blots are in Supplementary Fig. 5. Error bars represent standard deviation. Statistical analysis was performed using Kruskal–Wallis followed by Dunn's multiple comparison test. *ns* not significant, *p < 0.05, **p < 0.01, ***p < 0.001.

Exploring the therapeutic potential of SIRT3 activation, we investigated the effects of honokiol, a known SIRT3 activator, in a mouse model of CsA nephrotoxicity. Our findings demonstrated that honokiol administration significantly reduces serum creatinine and blood urea nitrogen levels in CsA-treated mice, indicating improved kidney function (Fig. 5A). We found SIRT3 activation effectively reduced the elevated levels of cleaved caspase-3 in vivo, aligning with our in vitro findings. Additionally, we examined the expression of mitochondrial dynamics-related genes, MFN1 and DRP1. While MFN1 expression remained unaffected, DRP1 levels, which decreased upon CsA treatment, experienced further reduction with SIRT3 activation (Fig. 5B). In histopathological analyses, tubular injury scores and fibrosis areas, along with TUNEL-positive cell counts, which were elevated in response to CsA, showed significant reductions with honokiol treatment (Fig. 5C).

## Discussion

Kidney injury induced by CsA was aggravated by downregulation of SIRT3 in both in vivo and vitro studies. The mitochondrial regulator protein pathway, AMPK-PGC1α, was estimated to be involved in the process of worsening injury by modulating oxidative stress. Furthermore, SIRT3 overexpression alleviated apoptosis in kidney tubular cells after CsA toxicity.

SIRT3 is an important member of sirtuin family, a conserved family of NAD + -dependent deacetylases, and has been studied for its role in various organs and diseases as potent deacetylase^17–19^. In heart, downregulation of SIRT3 increased sensitivity in ischemia reperfusion injury^17,18^. SIRT3 involved in insulin resistance and diabetes mellitus, and has protective effect in retina, skeletal muscle and heart injury by diabetes^15,19^. Additionally, SIRT3 reported to has a dual role in cancer, acting as either a tumor suppressor or promoter by regulating ROS levels in tumor cells^20^. These broad effects of SIRT3 are primarily associated with its defense mechanism against oxidative stress, which decreases intracellular ROS levels to promote cell survival in various injury conditions^21,22^.

CsA nephrotoxicity is mainly attributed to vascular constriction and changes in vascular resistance. CsA induces focal hypoxia and ischemia in the kidney tubulointerstitium, resulting in cellular injury and apoptosis by increasing ROS and free radicals^5,23^. In addition, CsA directly activates pro-apoptotic genes and induces tubular atrophy by increasing apoptosis of tubulointerstitial cells^24,25^. In this study, we confirmed that SIRT3 has significant role in CsA nephrotoxicity mediated by mitochondrial dysfunction, emphasizing the importance of mitochondrial homeostasis in CsA nephrotoxicity. With the potent acetylase activity of SIRT3, these effects seem to be accompanied by downregulation of AMPK and PGC1α, which are known as mitochondrial regulators. Mitochondrial ROS promotes AMPK activation and subsequently induces PGC1α activation which trigger antioxidative response. In previous studies, SIRT3 regulated mitochondrial biogenesis via PGC1α mediated ROS inhibition^21^. Furthermore, there are evidences that SIRT3 activate AMPK via various process including LKB1 and CaMKKβ^26,27^. In our research, the differential impacts observed from SIRT3 modulation—where its suppression via siRNA led to a decrease in PGC-1α without affecting p-AMPK, and its overexpression increased both markers—highlight SIRT3's intricate role in cellular energy dynamics. Suppression of SIRT3 underscores its critical function in mitochondrial integrity, directly influencing PGC-1α, a key mitochondrial biogenesis regulator. Conversely, SIRT3 overexpression suggests its capability to enhance mitochondrial function, indirectly activating AMPK, a central cellular energy sensor. This dual action of SIRT3, affecting both PGC-1α and AMPK, illustrates its broad regulatory scope within cellular energy metabolism and mitochondrial maintenance. Our findings underscore the need for further investigation into its regulatory mechanisms and their implications for mitochondrial function and cellular energy homeostasis.

To establish the pivotal effect of SIRT3 on CsA injury in context of mitochondrial homeostasis, we evaluated mitochondrial dysfunction by various methods including measurement of mitochondrial membrane potential and mitochondrial ROS, assessing expression of SOD2 in mitochondria, and analysis of genes associated with mitochondrial fusion and fission, along with the production of end products of kidney oxidation. SOD2 has been implicated in mitochondrial ROS clearance and has a capacity to limit the detrimental effects of ROS^28,29^. The tyrosine and fatty acid oxidation product, nitrotyrosine and hydroxynonenal, were used as an indicator of oxidative stress^30,31^. Our cellular experiments confirmed that upregulation of SIRT3, irrespective of CsA injury, increases the expression of SOD2. This observation suggests that SIRT3 plays a crucial role in enhancing the cell’s antioxidative defenses by upregulating SOD2, a key enzyme in detoxifying mitochondrial reactive oxygen species (ROS). In contrast, DRP1, which is involved in mitochondrial fission, was significantly reduced in the context of SIRT3 and CsA damage, and this reduction was further accelerated by the administration of a SIRT3 activator. Typically, excessive mitochondrial fission is observed under conditions of cellular stress and damage, which can promote apoptosis. The reduction of DRP1 following CsA injury could be a natural inhibitory response of mitochondrial fission to cellular damage. In alignment with our study, previous research has shown that down-regulation of DRP1 leads to mitochondrial elongation, reduced cell proliferation, and increased apoptosis in human colon cancer cells^32^. However, the further reduction of DRP1 by SIRT3 activation, while not intuitive, suggests that SIRT3 may act through a protective mechanism that supports mitochondrial quality control and cell survival. The additional decrease in DRP1 mediated by SIRT3 could further inhibit mitochondrial fission, delay the removal of damaged mitochondria, and promote the maintenance of a larger and more efficient mitochondrial network^33^. This process could enhance cellular energy efficiency and suppress apoptosis, ultimately providing protection against cell damage induced by CsA.

Meanwhile, our study has demonstrated the protective effect of SIRT3 against apoptosis by reducing various apoptosis markers, although not uniformly across all markers. Specifically, while cleaved caspase-3 and Bax significantly declined with SIRT3 overexpression, cytochrome c's reduction didn’t reach statistical significance. This variation may stem from the intricate apoptosis regulation mechanisms. SIRT3 directly affects upstream markers like Bax and caspase activation through enhancing antioxidant defenses and mitochondrial integrity. The influence on cytochrome c release, however, appears subtler, governed by factors beyond Bax's control, such as mitochondrial membrane potential and other mitochondrial proteins. Additionally, cytochrome c's variability may reflect the balance between pro- and anti-apoptotic signals, suggesting SIRT3's effects are more directly counteracting mechanisms leading to caspase and Bax activation rather than those controlling cytochrome c release. This suggests that while SIRT3 overexpression can significantly impact certain apoptosis markers, its effect on cytochrome c might be less direct or require additional factors to manifest significantly.

Although this study has the strength of being the first to confirm the role of SIRT3 in CsA nephrotoxicity, there are also limitations. Notably, the specific acetylation targets of SIRT3 within the context of CsA nephrotoxicity remain unidentified, which represents a crucial area for future research. Other processes implicated in CsA nephrotoxicity, such as the fas/fas-L related pathway, were not evaluated in this study. Additionally, the potential downstream or upstream effectors of SIRT3, such as AMPK and PGC1a, were not modulated. Further research is warranted to clarify the hierarchy of these proteins.

Overall, this study demonstrates the role of SIRT3 in CsA nephrotoxicity through the regulation of mitochondrial homeostasis, providing evidence for understanding the mechanisms underlying CsA nephrotoxicity. Further studies may be warranted to investigate the potential of SIRT3 as a target for the prevention or treatment of CsA nephrotoxicity.

## Methods

### Cell culture

Madin-Darby canine kidney cells (MDCK) were obtained from Korean Cell Line Bank (KCLB, Seoul, Korea). MDCK cells were cultured in Dulbecco Modified Eagle Medium (DMEM) with 10% fetal bovine serum (FBS, Gibco, Grand Island, NY, USA) at 37 °C with 5% CO_2_. For the cyclosporine toxicity model, Cells were treated with 20 uM of CSA contained with vehicle polyoxyethylated caster oil (Cremophor EL, Sigma Aldrich, St. Louis, MO, USA) Vehicle (Cremophor EL, Sigma) equivalent to the 20 uM was used as control.

### SIRT3 overexpression by Flag-tagged SIRT3 transfection

Flag-tagged SIRT3 containing plasmid with pcDNA3.1( +) backbone was purchased from Addgene (#13814, Cambridge, MA, USA). DH5α competent cells (Real Biotech Corporation, Taiwan) was transformed with Flag-SIRT3 plasmid and amplified with E.coli cultures. The plasmid DNA was purified by DNA-spin Plasmid DNA Purification Kit (#17099, iNtRON, Seongnam, Korea) according to the manufacturer’s instruction.

For overexpression of SIRT3, cells were transfected with pcDNA3.1 flag-SIRT3 plasmid and empty vector by using Lipofectamine 3000 reagent (Invitrogen, Carlsbad, CA, USA).

### SIRT3 inhibition by small interfering RNA transfection

Three SIRT3 siRNA (# 1, 2 and 3) were designed targeting partial dog SIRT3 mRNA sequence (XM_022405277.1) and constructed by Bioneer (Daejeon, Korea) (Supplementary Fig. 1).

To test the efficiency of the three SIRT3 siRNA, MDCK cells were transfected with SIRT3 siRNA by using Lipofectamine RNA iMAX reagent (Invitrogen). SIRT3 expression was evaluated by western blotting. The study was conducted with SIRT3 siRNA #3, the most effective siRNA: sense: 5′ CUGCCUCAAAGCUGGUUGA 3′, antisense: 5′ UCAACCAGCUUUGAGGCAG 3′. Negative control siRNA (Ambion, TX, USA) was used as control for off-target effects.

### Mitochondrial isolation

Mitochondria were isolated by using a mitochondria isolation kit (#89801, Thermo Fisher, Waltham, MA, USA). Briefly, pellet of harvested cells was lysed with reagent A and centrifuged at 700 g for 10 min at 4 °C. The supernatant was collected and added with reagent B and C. The mixture was centrifuged at 12,000*g* for 15 min at 4 °C. The obtained pellet was used as a fraction of mitochondria for western blot analysis.

### Western blot analysis

Harvested cells or tissues were lysed and lysates were quantified by performing bicinchoninic acid (BCA) assay (Thermo Fisher). SDS-PAGE was performed and then transferred onto polyvinylidene difluoride membranes. The membrane was incubated with the following primary antibodies : antibody against SIRT3 (LS-C31605, LS-Bio, Washington, USA), PGC1α (NBP1-04676, Novus Biologicals, Littleton, USA), AMPKα (#2532, Cell Signaling Technology, Danvers, MA, USA), p-AMPKα (#2535, Cell Signaling Technology,), SOD2 (sc-30080, Santa Cruz Biotechnology, Dallas, TX, USA), cytochrome C (#556433, BD Biosciences, San Diego, CA, USA), Bax, (#2772, Cell Signaling Technology), BcL-2 (#2876, Cell Signaling Technology), Caspase-3 (#9662, Cell Signaling Technology), β-actin (A5316, Sigma Aldrich), HSP60 (sc-13115, Santa Cruz Biotechnology), MFN1 (#13798-1-AP, Proteintech, China), DRP1 (#ab156951, Abcam, Cambridge, United Kingdom). The blots were incubated with following horseradish peroxidase-conjugated secondary antibodies: Anti-rabbit IgG (PI-1000, Vector Laboratories Inc., Burlingame, CA, USA) and anti-mouse IgG (PI-2000, Vector Laboratories Inc.) The immunoblots were visualized by enhanced chemiluminescence (ECL, Perkin Elmer, Waltham, MA, USA) and exposed on X-ray film (Agfa-Geavert, NV, Morstel, Belgium). Protein expression was quantified using Image J Program (National Institutes of Health, Bethesda, MD, USA). In our western blot analyses, due to the requirement to detect several molecules simultaneously, each membrane is strategically cut into sections. For this reason, we do not possess images of full-length membranes for each antibody used.

### Cell viability assay

Cell viability was determined using Cell Titer 96 Aqueous One solution Cell proliferation Assay Kit (G3580, Promega, Madison, WA, USA). Cells were seeded into 96 well plate and treated with CsA. Cells were stained with 20 uL of 3-(4, 5-dimethylthiazol-2-yl)-5-(3-carboxymethoxyphenyl)-2-(4-sulfophenyl)-2H-tetrazolium, inner salt (MTS) for 1 h at 37 °C. The absorbance was estimated with microplate reader (SpectraMAX, 190, Molecular Devices, CA, USA) at 490 nm.

### Mitochondrial membrane potential test

Mitochondrial membrane potential were measured using 5,5′,6,6′-tetrachloro 1,1′,3,3′-tetraethylbenzimidazolylcarbocyanine iodide (JC-1) (T3168, Thermo Fisher) by flow cytometry, according to the manufacturer’s instructions. Briefly, 3 × 10^5^ cells were seeded and stained with JC-1 dye for 20 min at 37 °C. With the flow cytometer (BD LSR Fortessa X-20, BD Biosciences), green JC-1 monomers in apoptotic cells were detected in FL1 channel (525 nm) and red JC-1 monomers aggregates in healthy cells were detected in FL2 channel (590 nm).

### Mitochondrial ROS measurement

Mitochondrial ROS was detected by flow cytometry of labeling cells with MitoSOX Red mitochondrial superoxide indicator (M36008, Invitrogen). In brief, 3 × 10^5^ cells were seeded and stained with 5 μM of MitoSOX Red for 20 min at 37 °C. The stained cells were excited at 510 nm and the emitted fluorescence was detected at 580 nm by flow cytometry (BD LSR Fortessa X-20, BD Biosciences). The measurements were independently repeated for three times.

### Animal study

All experimental protocols and animal handling procedures were performed according to the National Institutes of Health (NIH) guidelines for the use of experimental animals, and the study was approved by the Korea University Institutional Animal Care and Use Committee (IACUC Approval no : KOREA-2020-0040-C3). This study is reported in accordance with ARRIVE guidelines. We purchased SIRT3 heterozygous knockout mice (B6.129S6(Cg)-Sirt3tm1.1Fwa/J) from Jackson Laboratory, and mated them and checked genotypes to gain homozygous SIRT3 KO mouse. For the control, weight and age-matched C57BL/6 J mice were purchased. All experiments were conducted using 8-week old mice weighing 20-25 g each. To induce cyclosporine nephrotoxicity, mice were feeded with low sodium diet (AIN-76A Basil Sodium deficient diet) and allowed free access to water^34,35^. Mice in cyclosporine toxicity group were subcutaneously injected one in a day 100 mg/ml of cyclosporine diluted in olive oil as 30 mg/kg of final concentration for 4 weeks. And the mice in control group were injected olive oil once a day with same volume as in treatment group for 4 weeks. For SIRT3 activation experiments, honokiol was administered to investigate its SIRT3 activation potential^36,37^. Honokiol treatment commenced simultaneously with cyclosporine administration at a dosage of 0.2 mg/kg/day and was maintained throughout the 4-week course of the study. After 4 weeks of experimentation, serum and kidney tissues were harvested under anesthesia, which was induced with 3–4% isoflurane and maintained at 1–3% isoflurane. Following the collection of tissues, mice were euthanized via cervical dislocation. Serum blood urea nitrogen and serum creatinine were measured by biochemistry analyzer (Fuji Dry-Chem NX500i, Fujifilm, Kanagawa, Japan).

### Histology, immunohistochemistry and TUNEL stain

Periodic acid-Schiff base (PAS) staining and Masson’s trichrome staining were performed to evaluate the severity of inflammation and fibrosis on kidney. Tubular injury was scored as follows: 0, normal; 1, minimal (≤ 10% involvement); 2, mild (10–25% involvement); 3, moderate (26–50% involvement); 4, severe (51–75% involvement); and 5, very severe (> 75% involvement). Injured area determined by tubular atrophy or dilatation, brush border loss, vacuolization, and nuclear condensation. The area of fibrosis was quantified by measuring the stained regions in Masson’s trichrome-stained sections.

For the immunohistochemical assays, paraffin-embedded kidneys which were cut into 4 µm-thick slices were deparaffinized and hydrated using xylene and ethanol. After peroxidase blocking and antigen retrieval, the deparaffinized sections were stained with 4-Hydroxynonenal antibody (1:4000, Bioss), nitrotyrosine antibody (1:400, Invitrogen) and α-smooth muscle actin antibody (1:200, cell signaling technology) and then incubated with horseradish peroxidase-conjugated goat anti-rabbit IgG (Vector Laboratories, Burlingame, CA, USA), respectively. After counterstained with Mayer’s hematoxylin (Sigma-Aldrich), mounted slides were evaluated under a light microscope.

Apoptosis in kidney tissues was determined using a TUNEL assay with an ApopTag^®^ Peroxidase In Situ Apoptosis Detection Kit (Sigma-Aldrich). The number of TUNEL-positive cells was counted in 10 random high power fields.

### Statistical analysis

All statistical analyses were performed using GraphPad Prism 7.0 software (Graph Pad, Inc., La Jolla, CA, USA). Data were expressed as the means ± standard deviation or means ± standard error of the mean, wherever indicated. A p value of < 0.05 was considered statistically significant.

### Supplementary Information

Supplementary Figures.

## Data Availability

The datasets used and/or analyzed during the current study are available from the corresponding author on reasonable request.
