# Supplementary material for: Mitochondrial SIRT3 as a protective factor against cyclosporine A-induced nephrotoxicity
Source: Sci Rep. 2024 May 2;14:10143. doi: 10.1038/s41598-024-60453-4 (PMC11065982; doi:10.1038/s41598-024-60453-4)
Supplement: Supplementary file 1 — Supplementary Figures. [file 41598_2024_60453_MOESM1_ESM.pptx]

## Slide 1
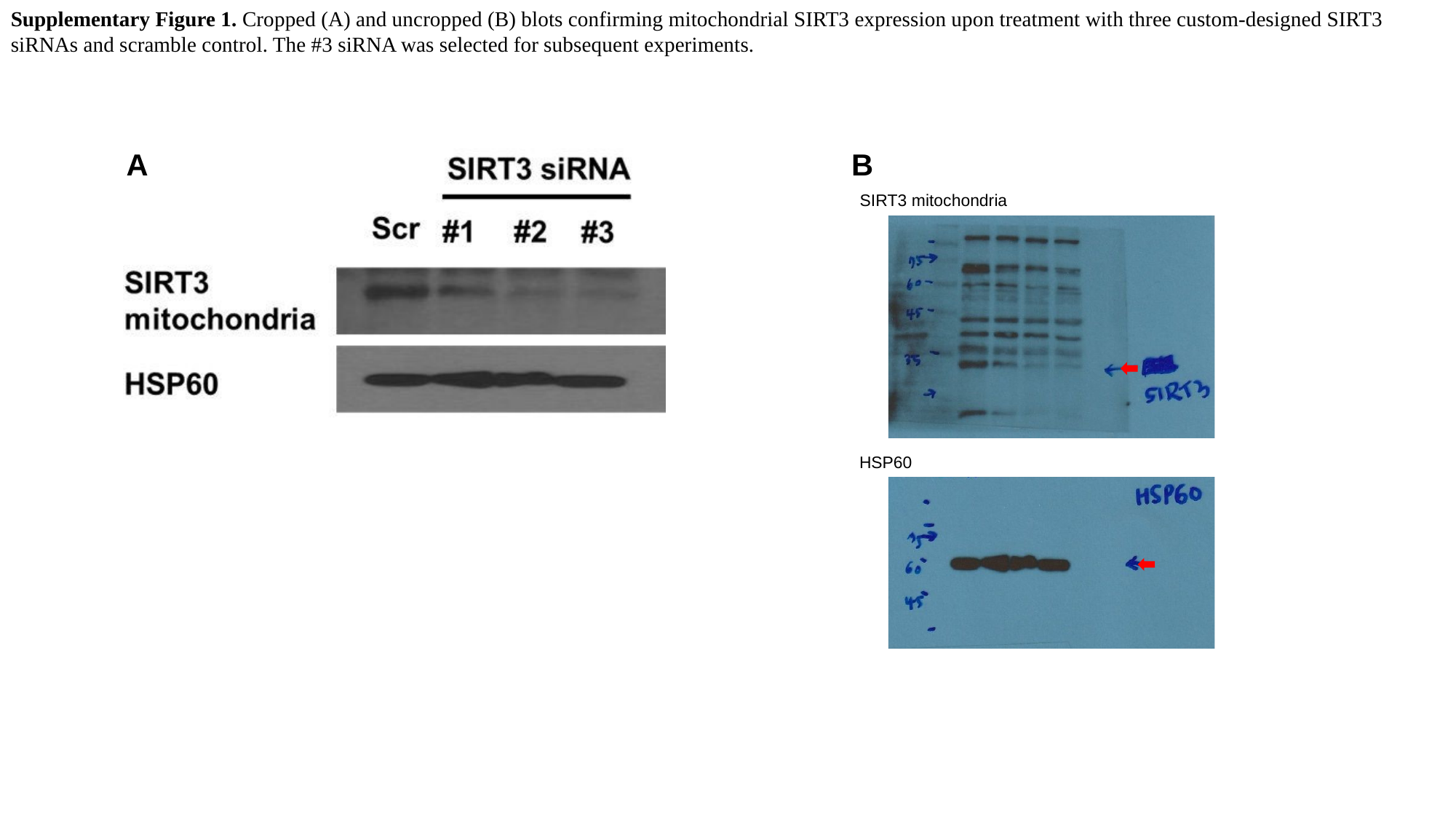

Supplementary Figure 1. Cropped (A) and uncropped (B) blots confirming mitochondrial SIRT3 expression upon treatment with three custom-designed SIRT3 siRNAs and scramble control. The #3 siRNA was selected for subsequent experiments.
A
B
SIRT3 mitochondria
HSP60

## Slide 2
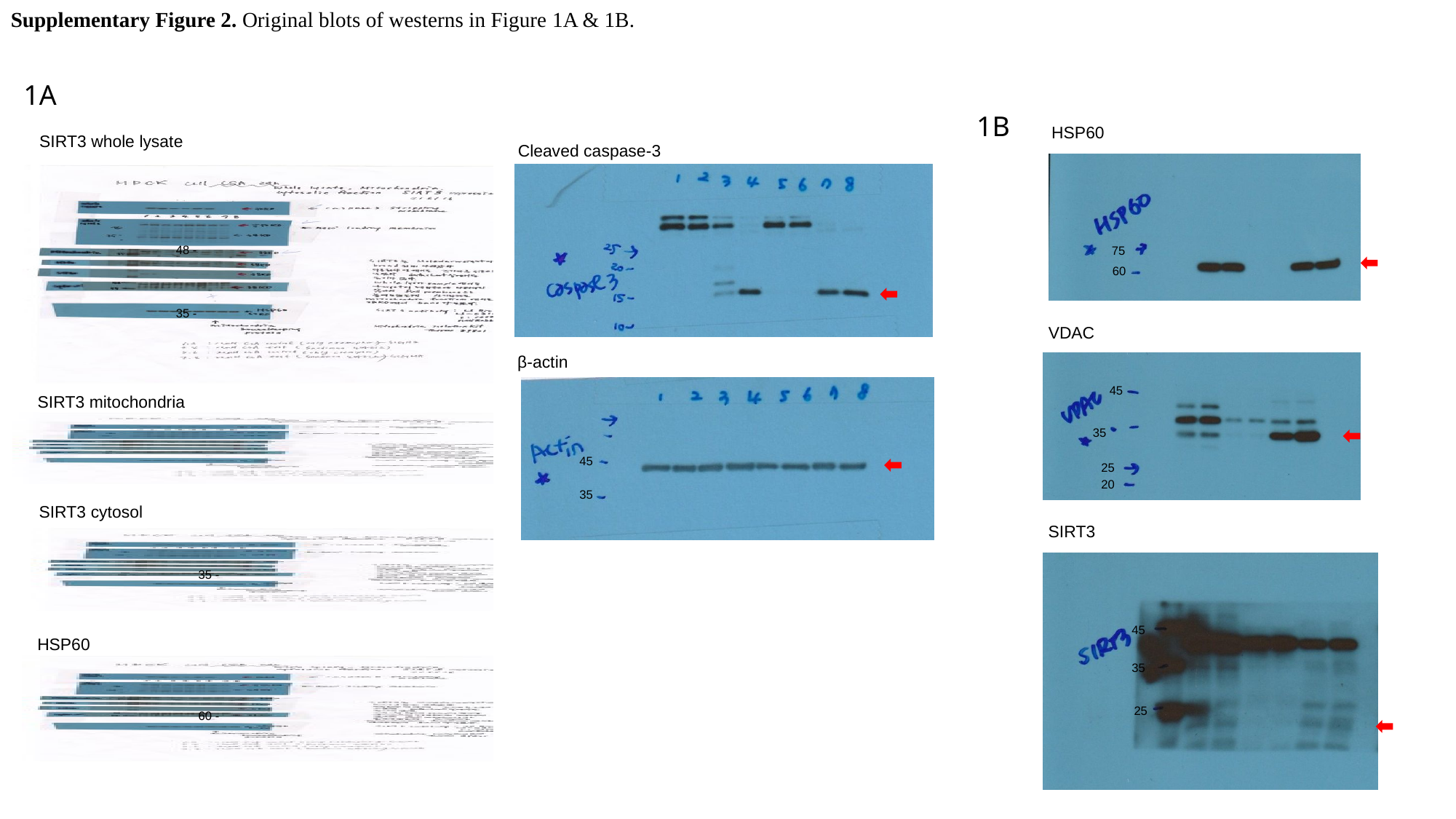

Supplementary Figure 2. Original blots of westerns in Figure 1A & 1B.
1A
1B
HSP60
SIRT3 whole lysate
Cleaved caspase-3
48 -
75
60
35 -
VDAC
β-actin
45
SIRT3 mitochondria
35
45
25
20
35
SIRT3 cytosol
SIRT3
35 -
45
HSP60
35
25
60 -

## Slide 3
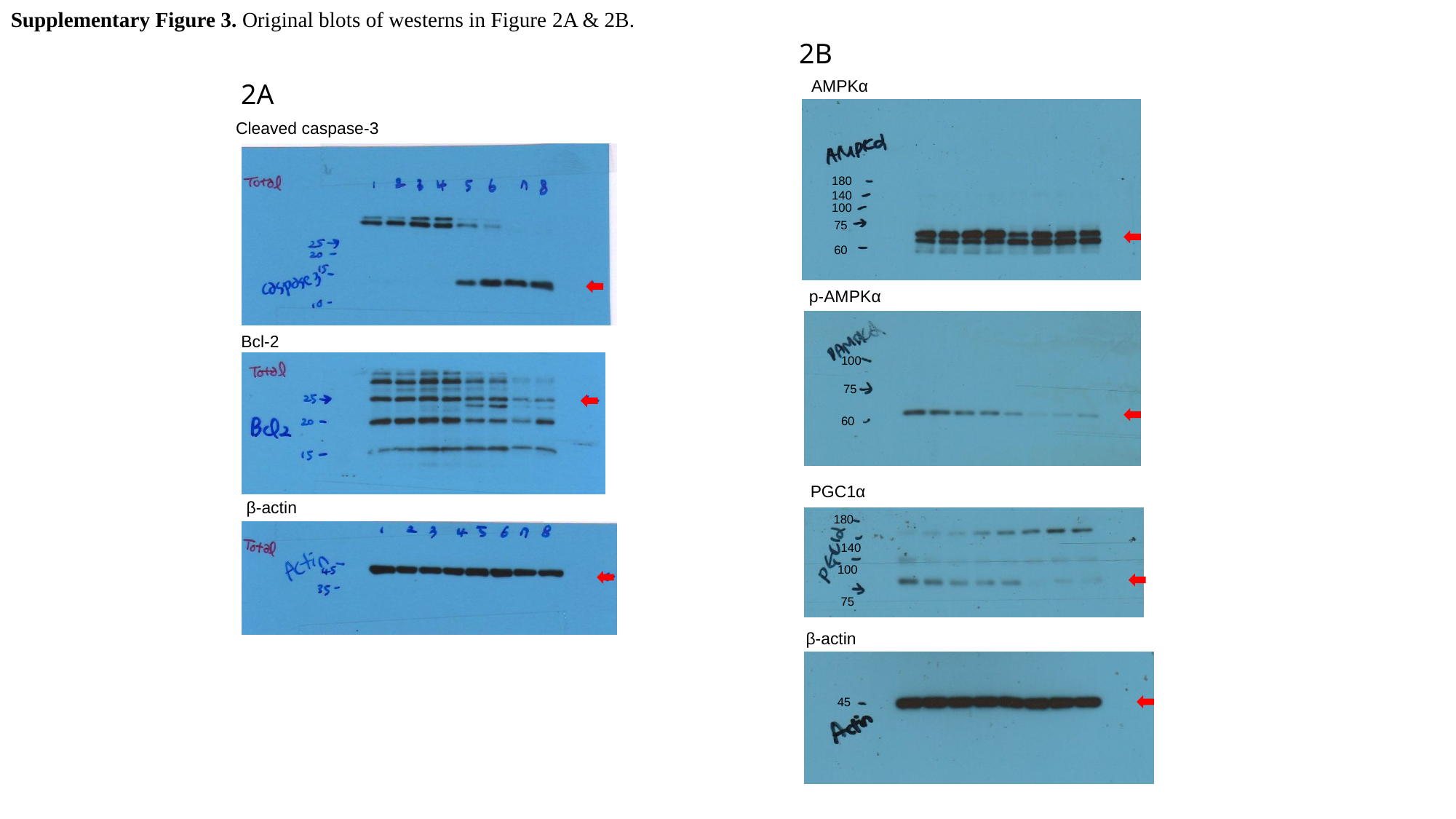

Supplementary Figure 3. Original blots of westerns in Figure 2A & 2B.
2B
AMPKα
2A
Cleaved caspase-3
180
140
100
75
60
p-AMPKα
Bcl-2
100
75
60
PGC1α
β-actin
180
140
100
75
β-actin
45

## Slide 4
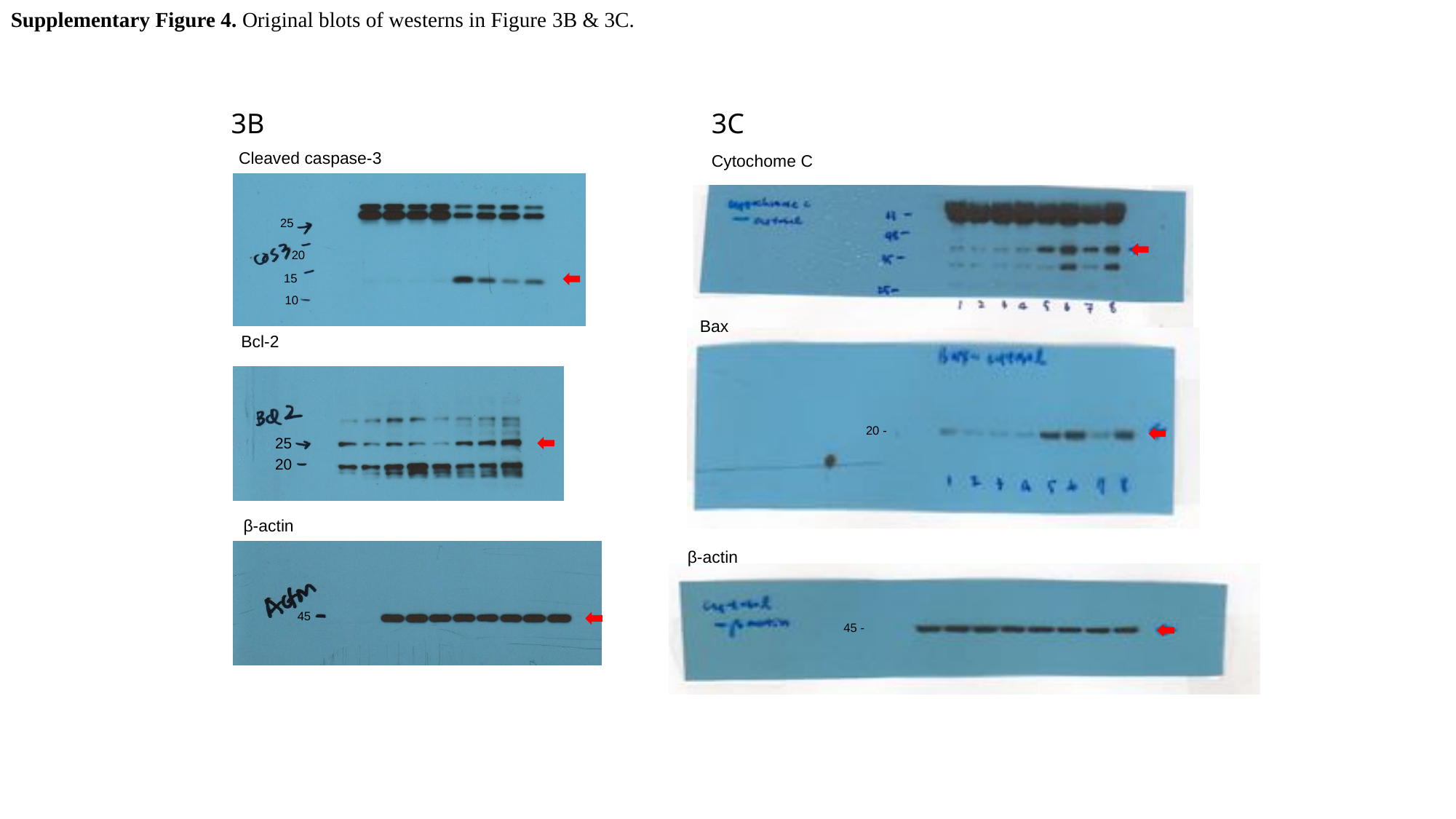

Supplementary Figure 4. Original blots of westerns in Figure 3B & 3C.
3B
3C
Cleaved caspase-3
Cytochome C
25
20
15
10
Bax
Bcl-2
20 -
25
20
β-actin
β-actin
45
45 -

## Slide 5
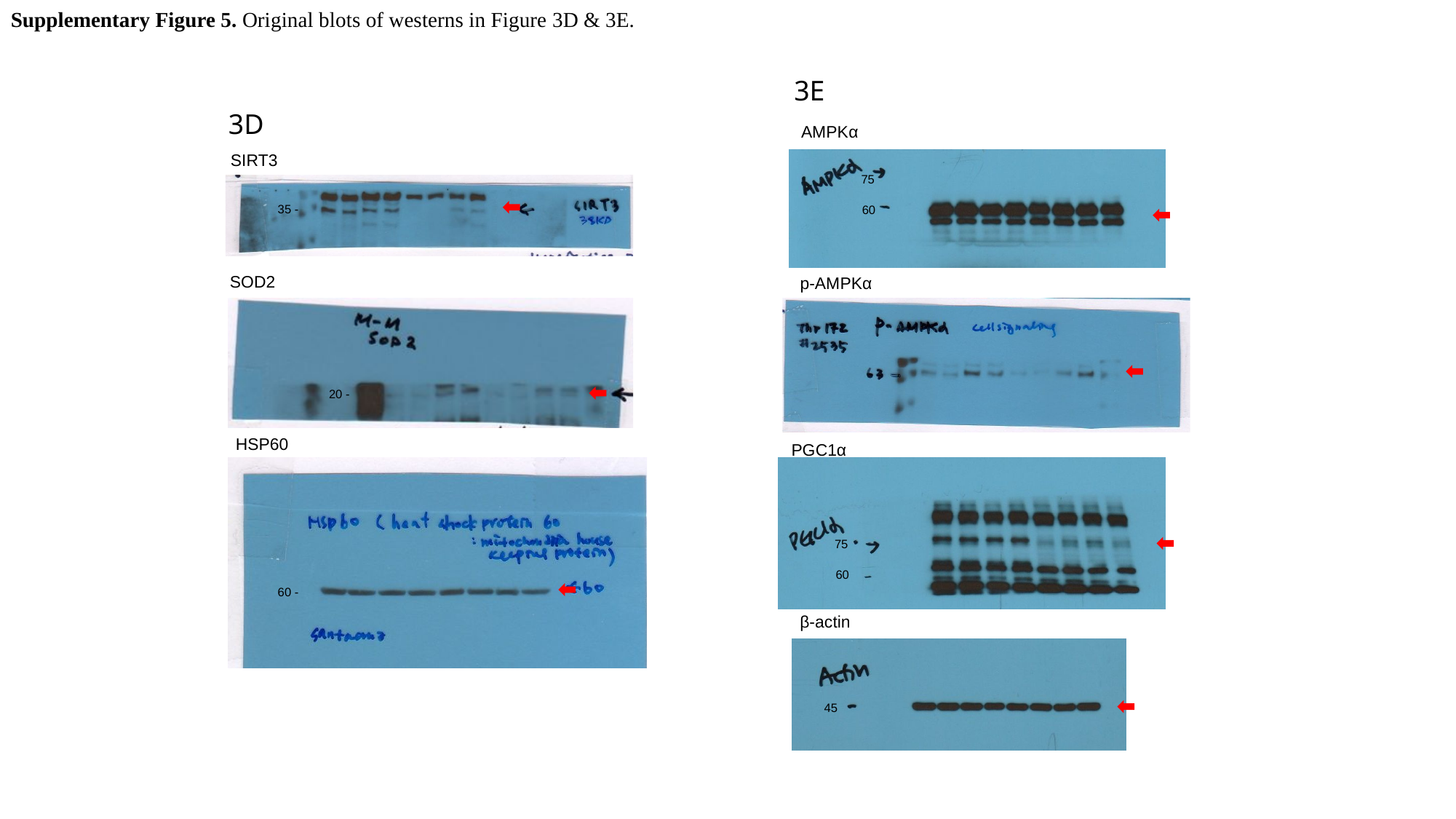

Supplementary Figure 5. Original blots of westerns in Figure 3D & 3E.
3E
3D
AMPKα
SIRT3
75
35 -
60
SOD2
p-AMPKα
20 -
HSP60
PGC1α
75
60
60 -
β-actin
45

## Slide 6
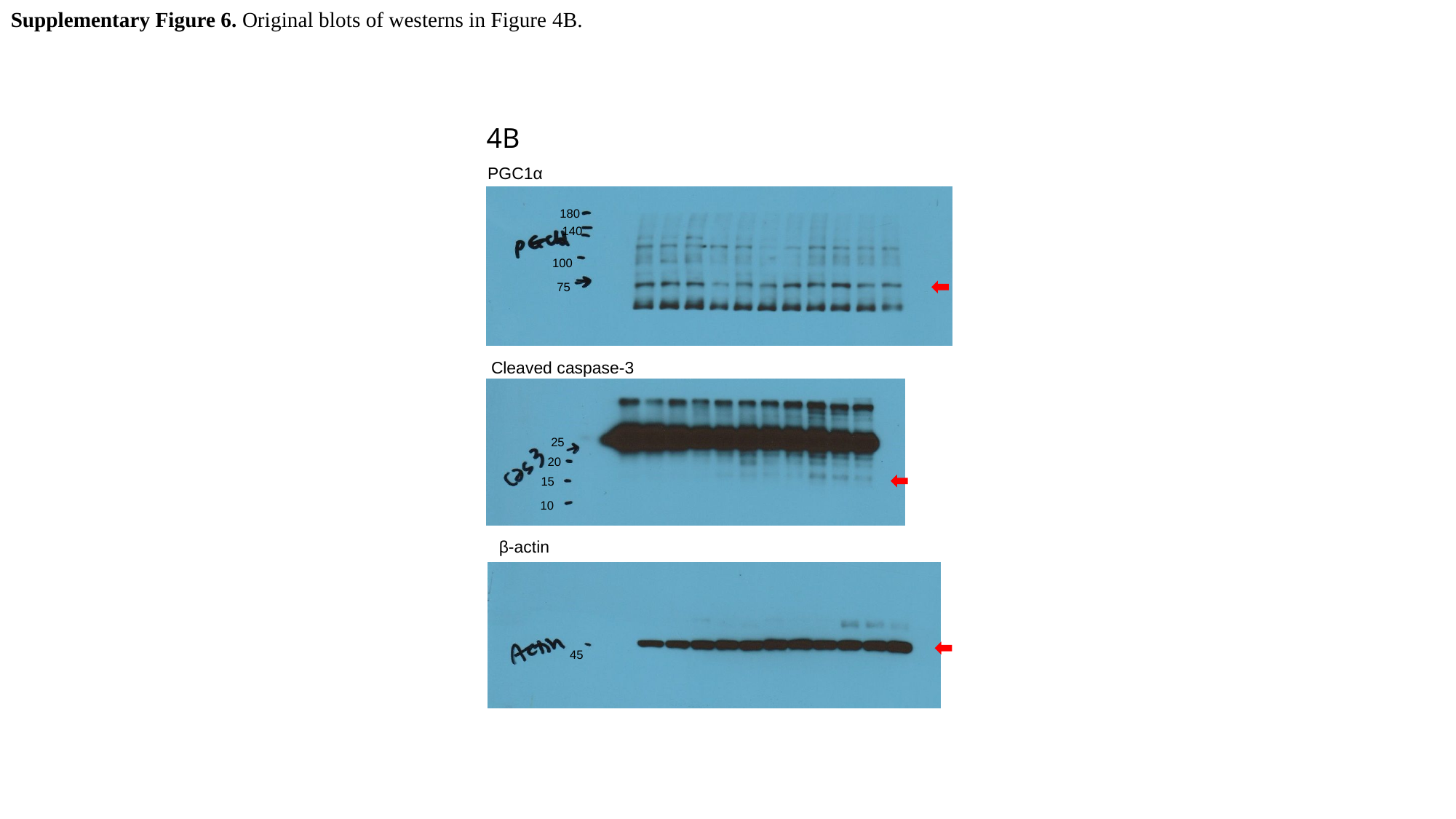

Supplementary Figure 6. Original blots of westerns in Figure 4B.
4B
PGC1α
180
140
100
75
Cleaved caspase-3
25
20
15
10
β-actin
45

## Slide 7
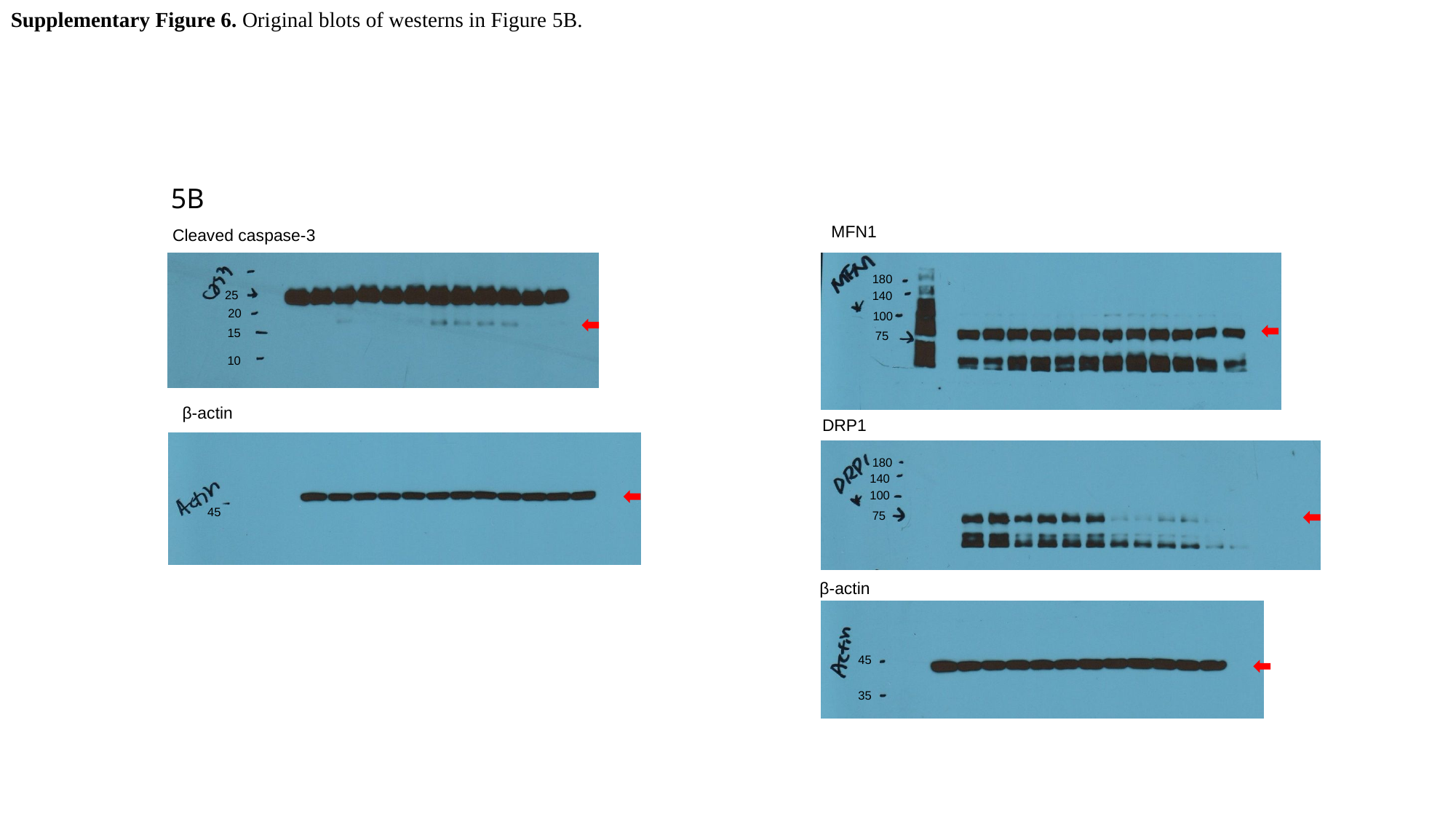

Supplementary Figure 6. Original blots of westerns in Figure 5B.
5B
MFN1
Cleaved caspase-3
180
25
140
20
100
15
75
10
β-actin
DRP1
180
140
100
45
75
β-actin
45
35
